# The Presence of Gut Microbial Genes Encoding Bacterial Genotoxins or Pro-Inflammatory Factors in Stool Samples from Individuals with Colorectal Neoplasia

**DOI:** 10.3390/diseases7010016

**Published:** 2019-02-01

**Authors:** Ramón Gómez-Moreno, María González-Pons, Marievelisse Soto-Salgado, Marcia Cruz-Correa, Abel Baerga-Ortiz

**Affiliations:** 1 University of Puerto Rico Medical Sciences Campus, San Juan 00936, Puerto Rico; ramon.gomez@upr.edu (R.G.-M.); maria.gonzalez9@upr.edu (M.G.-P.); marievelisse.soto1@upr.edu (M.S.-S.); marcia.cruz1@upr.edu (M.C.-C.); 2 Molecular Sciences Research Center, San Juan 00926, Puerto Rico; 3 University of Puerto Rico, Comprehensive Cancer Center, San Juan 00936, Puerto Rico

**Keywords:** gut microbiota, colorectal neoplasia, colorectal cancer, microbial biomarkers

## Abstract

Gut bacterial toxins are thought to contribute to the development of colorectal cancer (CRC). This study examines the presence of specific gut bacterial toxin genes in stool samples from individuals with colorectal neoplasia (adenomas and/or CRC). The presence of bacterial genes encoding genotoxic or pro-inflammatory factors (*pks*, *tcpC*, *gelE*, *cnf-1*, *AMmurB*, and *usp*) was established by PCR of stool samples from individuals from mainland US (*n* = 30; controls = 10, adenoma = 10, CRC = 10) and from Puerto Rico (PR) (*n* = 33; controls = 13; adenomas = 8; CRC = 12). Logistic regression models and multinomial logistic regression models were used to estimate the magnitude of association. Distinct bacterial gene profiles were observed in each sample cohort. In individuals with CRC, *AMmurB* was detected more frequently in samples from the US and *gelE* in samples from PR. In samples from PR, individuals with ≥2 gut bacterial toxin genes in stool had higher odds of having colorectal neoplasia (OR = 11.0, 95%: CI 1.0–637.1): however, no significant association between bacterial genes and colorectal neoplasia was observed in the US cohort. Further analyses are warranted in a larger cohort to validate these preliminary findings, but these encouraging results highlight the importance of developing bacterial markers as tools for CRC diagnosis or risk stratification.

## 1. Introduction

Sporadic, non-hereditary colorectal cancer (CRC) is a complex and multifactorial disease involving genetic, environmental, and lifestyle risk factors. CRC survival is largely dependent on prevention and early detection [1,2]. Currently, routine CRC screening and removal of adenomas (pre-cancerous lesions) are the primary means for prevention; however, the fact that 61% of CRC patients are diagnosed at more advanced, less treatable stages emphasizes the need for risk-stratified CRC prevention strategies that incorporate the individual’s modifiable and non-modifiable risk factors [2].

One of the most currently studied environmental risk factors associated with CRC is the gut microbiota [3,4]. Compared to healthy subjects, individuals with CRC have been reported to have a distinct gut microbiota composition enriched in gram-negative bacteria [5,6], which may include opportunistic pathogens such as *Escherichia* and *Campylobacter* that may harbor toxins that induce DNA damage, genomic instability, inflammation, and aberrant cell signaling and other hallmarks of cancer [7,8,9,10,11]. Accumulating evidence supports the notion that a subset of the gut microbiota can promote CRC development through chronic inflammation and genotoxicity, among other possible pathways [12,13,14,15,16,17,18,19,20]. However, the precise mechanisms by which the gut microbiota exerts its CRC-promoting effects are still not fully understood. 

Although numerous studies suggest the involvement of individual gut bacterial species in the etiology of CRC, causality is yet to be established [3,10,14,21,22]. Recent studies have reported that genotoxin-producing *E. coli* strains are more prevalent in CRC [8,23,24] and that CRC tissues have more mucosa-associated *E. coli* than seemingly healthy tissues [25]. These findings support the idea that bacteria with genotoxic and/or pro-inflammatory toxins are not only more abundant in CRC, but that they are also in close proximity to the colonic epithelium where they can exert their pro-carcinogenic effects. 

In this study, we have assembled a panel of genes that includes six specific, genotoxic and/or pro-inflammatory gut bacterial genes that encode toxins with known pathogenic mechanisms, that may contribute to colorectal carcinogenesis (Table 1). Previously, our group reported a method for the detection of six gut bacterial toxin genes in DNA isolated from clinical stool samples [26]. In the present study, we report the association between the presence of these six gut bacterial toxin genes in stool and colorectal neoplasia using samples from two different geographical locations (mainland US and PR). 

## 2. Materials and Methods

### 2.1. Stool Sample Collection

Human stool samples were obtained from the Early Detection Research Network (EDRN; https://edrn.nci.nih.gov/) and the Puerto Rico Familial Colorectal Cancer Registry (PURIFICAR; http://purificar.rcm.upr.edu/ index_eng.html). The EDRN, an initiative of the National Cancer Institute (NCI), brings together dozens of institutions to help accrue biospecimens, accelerate the translation of biomarker information into clinical applications, and to evaluate new diagnostic tests for cancer. PURIFICAR is an island-wide registry that recruits healthy individuals and those with colorectal neoplasia. All subjects recruited by PURIFICAR complete the Colon Cancer Family Registry risk factor questionnaire, which collects sociodemographic and clinical information including: medical history, body mass index (BMI), lifestyle, family history of cancer, and demographic information, among others. PURIFICAR was approved by the University of Puerto Rico Institutional Review Board (approval number: A2210207).

The EDRN kindly provided 30 age- and gender-matched stool samples from individuals residing in the mainland US (controls = 10; adenoma = 10; and CRC = 10). Samples from individuals living in PR were obtained through PURIFICAR (*n* = 33; controls = 13; adenoma = 8; and CRC = 12), an island-wide population-based registry that collects biospecimens (blood, colorectal tissue, and stool) from both cases (individuals with colorectal neoplasia) and controls (healthy individuals without prior history of colorectal neoplasia). Only individuals with pathologic confirmation of adenomas and CRC were included in this study. Individuals diagnosed with Crohn’s disease or ulcerative colitis, that have undergone previous subtotal or total colectomies, or have had antibiotic treatment at any time in the three months previous to recruitment were excluded. All stool samples obtained through PURIFICAR were collected during a six-month period and stored at −80 °C.

### 2.2. Bacterial DNA Extraction

Bacterial DNA was extracted from human stool samples (200 mg/per sample) using the QIAamp^®^ DNA Stool Mini Kit (QIAGEN) according the manufacturer’s instructions. DNA extractions were performed within a one-month period since stool samples were received at the laboratory. A total of 5 μL of DNA extract was used as template for subsequent PCR analyses. DNA concentrations were determined using a Nanodrop (ThermoFisher Scientific) and total bacterial DNA was quantified using the formula: μg of DNA = A260 × 0.05 × Vol, where A260 is the absorbance at 260 nm and Vol is the total volume of eluted DNA in microliters.

### 2.3. PCR Profiling of Specific Bacterial Toxin Genes

The detection and quantification of the six gut bacterial toxin genes in our panel was carried out by PCR analyses as previously described [26]. DNA extracts from bacterial isolates known to contain the genes of interest were used as positive controls. The strains used as positive controls for the *pks*, *tcpC*, and *cnf* qPCRs were selected from a collection of *E. coli* clinical isolates that were part of a nosocomial infection surveillance study [34]. The *E. faecalis* strain H32, an isolate previously known to contain *gelE*, was kindly donated by Dr. Luis Ríos-Hernández from University of Puerto Rico-Mayagüez. *A. muciniphila* genomic DNA was purchased from the American Type Culture Collection. 

Gut bacterial toxin gene primer sequences, annealing temperatures, and expected PCR products are summarized in Table 2 and Table 3. Due to the limited amount of stool sample from individuals from the mainland US, only end-point PCR was used to detect the bacterial toxin genes. Briefly, an initial denaturation step of 1 min at 94 °C was performed followed 30 s at 94 °C, 30 s at the corresponding annealing temperature, and 3 min at 68 °C. All reactions were finalized with a final extension step of 10 min at 72 °C. *pks*, *tcpC*, *gelE*, *cnf-1*, *AMmurB*, and *usp* amplicons were sequenced to ensure primer specificity. Samples positive for any of the gut bacterial toxin genes in our panel were further analyzed to by qPCR quantify the corresponding gene copy number. 

**Figure A1:**
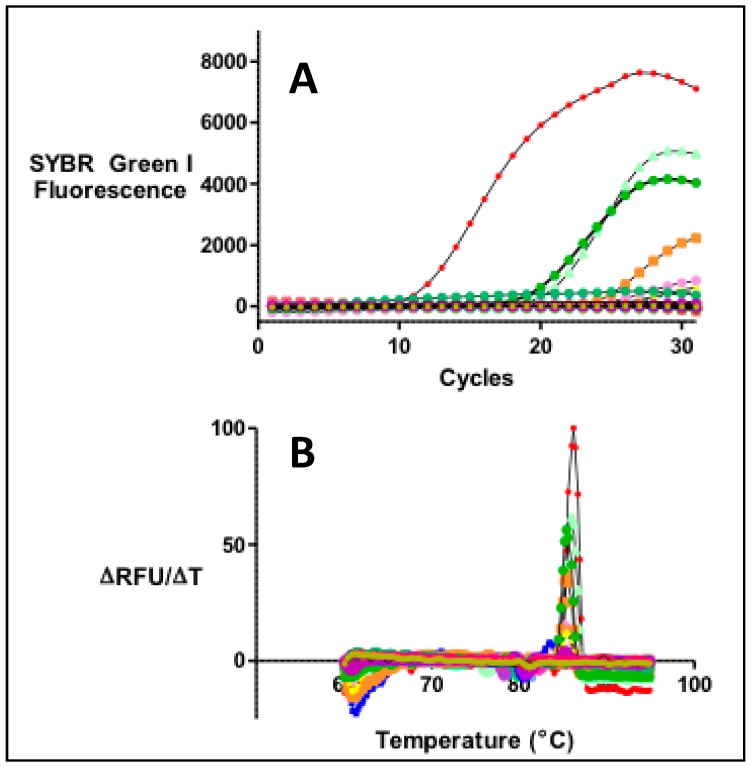
(**A**) Real-time PCR reactions were performed for the quantitation of DNA in stool samples compared with a set of standards of known concentration for each of the genes, (**B**) The presence of a single PCR product was ascertained by monitoring a DNA melting profile immediately after the PCR reaction. The presence of a single peak for each sample is indicative of a single PCR product.

Stool samples from individuals living in PR served as a validation set. The gut bacterial toxin genes in these samples were detected by qPCR. Detection of *pks*, *tcpC*, *gelE*, *cnf-1*, *AMmurB*, and *usp* in stool was performed in triplicate by qPCR analysis using the QuantiTect SYBR Green PCR kit (QIAGEN). Briefly, qPCR reactions required a 15 min incubation at 95 °C, followed by 50 cycles (for quantifying *gelE*) or 30 cycles (for the other genes in the panel) of 30 s at 94 °C, 30 s at the corresponding annealing temperature, and 3 min at 68 °C. All reactions had a final extension step of 10 min at 72 °C to ensure maximum detection. The presence of a single DNA amplicon at the end of the qPCR cycle was ascertained by a melting curve with a single-phase transition (Figure A1). Two independent qPCR assays were performed independently. Only the samples that tested positive in both independent measurements were counted as true positives (86% of all positives).

**Figure A2:**
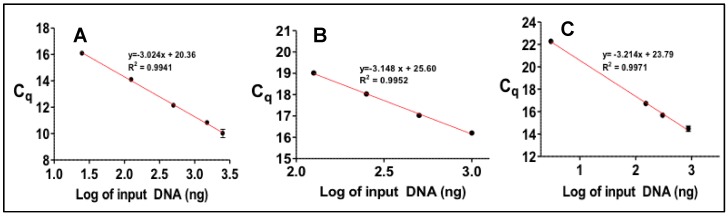
For the quantitation of DNA by real-time PCR a number of standards of known concentration were analyzed. The results for each gene was expressed as a function of average number of cycles (Cq) vs. known DNA concentration. Results are shown for (**A**) *pks island*, (B) *usp* and (**C**) *cnf-1*.

Standard curves using DNA extracts from bacterial isolates known to contain the bacterial toxin genes (Figure A2) were generated for the quantification of the gene copy numbers in our panel. All qPCRs reactions were performed as described in the section above. Gene copy numbers were calculated in stool samples for the bacterial toxin genes of interest. To calculate the number of gene copies in each sample, the DNA copy number conversion is determined from a measurement of absorbance at 260 nm, according to the following relationship:

gene copies = A260 × (50 × 10^−9^ gDNA/μL)/(GW)(1) (1)

where A260 is the absorbance of the sample at 260 nm and GW is the weight of the entire genome of the organism harboring the gene (5.1 × 10^−15^ g for *E. coli*, 3.6 × 10^−15^ g for *E. faecalis*, and, 2.7 × 10 × 10^−15^ g for *A. muciniphila*). The *E. coli* genome weight is used to calculate *pks*, *tcpc, cnf-1*, and *usp* gene copy numbers. *E. faecalis* and *A. muciniphila* genome weights are used to calculate *gelE* and *AMmurB* copy numbers, respectively.

### 2.4. Statistical Analysis

Logistic regression models were used to estimate the magnitude of association (Odds ratio with 95% confidence interval, OR with 95% CI) between colorectal neoplasia (CRC and polyps) and bacterial genes. In addition, multinomial (polytomous) logistic regression models were fitted to estimate the OR’s with 95% CI for CRC (outcome 1) and adenomas (outcome 2) compared with controls. The multinomial logistic regression was used to predict the probabilities of the different possible outcomes (CRC or adenomas) of a categorically distributed dependent variable given a set of independent variables (bacterial genes). All data was analyzed using Stata for Windows release 14.0 (Stata Corporation, College Station, TX, USA).

## 3. Results

### 3.1. Detection of Genotoxic or Pro-Inflammatory Bacterial Genes by PCR

**Figure 1:**
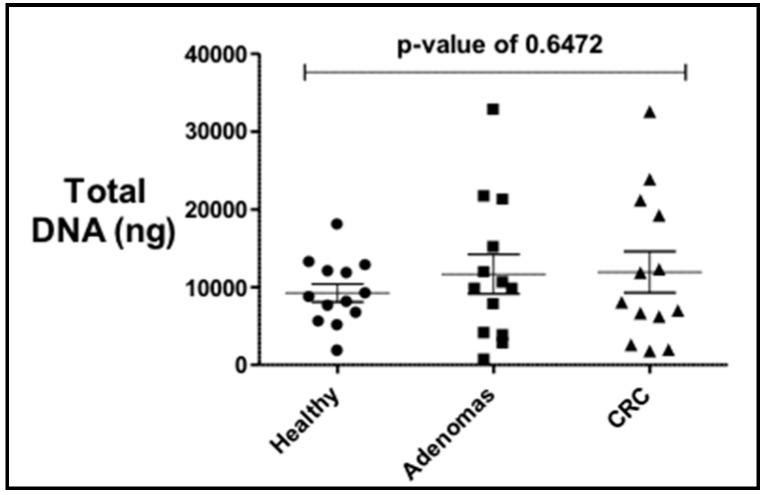
Total bacterial DNA extracted from stool samples of controls, adenomas and colorectal cancer (CRC) individuals shows no statistical difference by diagnosis. There is a higher variability in the adenomas and CRC.

**Figure 2:**
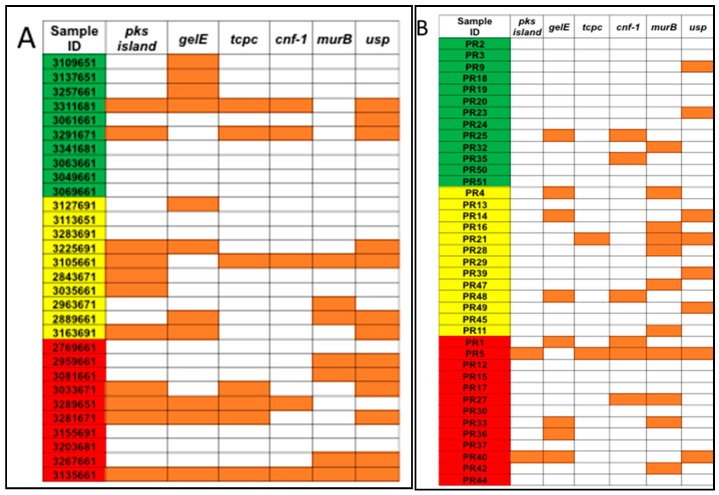
Pro-inflammatory and/or genotoxic bacterial genes were detected for (**A**) US samples from the Early Detection Research Network (EDRN) and (**B**) Puerto Rico samples from the University of Puerto Rico Comprehensive Cancer Center (UPR CCC). Samples were divided by diagnosis as controls (green), adenomas (yellow), and CRC (red). Orange squares represent samples that were positive for the gene.

The total bacterial DNA extracted ranged between 0.5 μg and 32 μg per 200 mg per stool sample. No statistical difference in the average total bacterial DNA concentrations between controls (9.2 μg), adenoma (11.6 μg), and CRC groups (11.9 μg) and the stool sample subgroups (US and PR) was observed (Figure 1). PCR analyses of stool samples from both mainland US and PR showed a higher frequency of genotoxic or pro-inflammatory toxin genes detected in samples from individuals with adenoma and CRC compared to controls (Figure 2). Also, differences were observed in the gut bacterial toxin gene profiles between samples from these two subgroups. In samples from the US, all of the gut bacterial toxin genes in our panel, except *AMmurB*, were detected in samples from healthy controls (Figure 2A). In the healthy group from PR, 4 of 6 of the gut bacterial toxin genes in our panel (*pks*, *tcpC*, *cnf-1*, and *AMmurB*) were detected (Figure 2B). In samples from PR with adenoma and CRC, *AMmurB* (50%) was more frequently detected in samples from individuals with adenomas and *gelE* (42%) in CRC samples. In the US cohort, *pks* (50%) was more frequently detected in samples from individuals with adenomas and *usp* (60%) in CRC stool samples.

The presence of genotoxic and/or pro-inflammatory bacterial toxin genes was associated with colorectal neoplasia in both of the study groups (US and PR cohorts); however, the degree of the association and the bacterial toxin genes associated varied between the two populations. Among the US cohort (Table 4), the odds of having CRC among individuals with *AMmurB* gene in their stool were 14.5-times (OR = 14.5, 95% CI: 0.7–316.7) compared to controls. However, this association was marginally significant (*p* = 0.09). No statistically significant associations between the presence of the gut bacterial toxin genes and colorectal neoplasia (adenoma and CRC) were observed in stool samples from individuals from the US. In the PR cohort, the presence of *gelE* in stool was marginally associated with adenomas (OR = 8.6, 95% CI: 0.8–89.04) (Table 5). In addition, the presence of ≥2 bacterial toxin genes in stool was significantly associated with adenomas (OR = 24; 95% CI: 1.11–518.6) and colorectal neoplasia (OR = 11.3; 95% CI: 1.0–637.1) compared to controls.

### 3.2. Gene Copy Number Measurements

**Figure 3:**
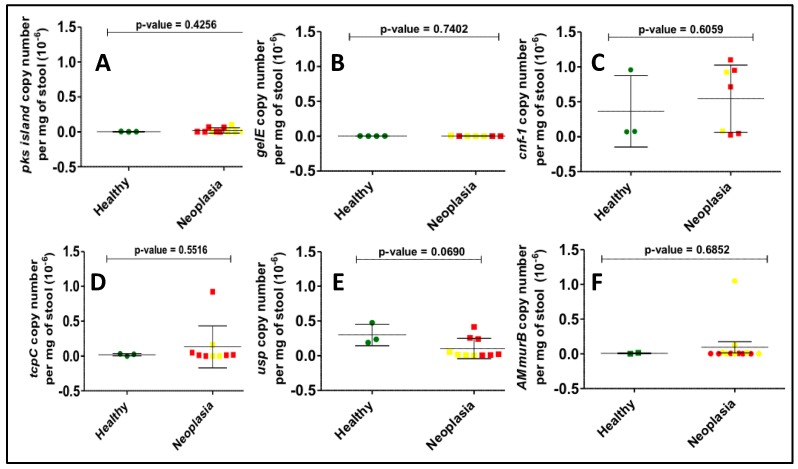
Determination of gene copy number per mg of stool for all positives among the US samples by real-time quantitative PCR. Here we show the results for (**A**) *pks island*, (**B**) *gelE*, (**C**) *cnf-1*, (**D**) *tcpC*, (**E**) *usp*, (**F**) *AMmurB* showing no statistical differences between the healthy (green dots) and neoplasia groups, which include adenomas (yellow dots) and CRC (red dots) individuals.

Bacterial toxin gene copy numbers were calculated in all samples positive for the bacterial genes in our panel. Although colorectal neoplasia samples were expected to have a higher bacterial gene copy number compared to the corresponding control samples, no differences were observed between the mean copy number of any of the bacterial genes studied (Figure 3). A higher degree of variability was observed for *usp* and *pks* gene copy numbers than for other genes.

## 4. Discussion

The gut microbiota has emerged as a major contributor to gastrointestinal carcinogenesis [35,36,37]. Despite new insights about the relationships between the gut microbiota and CRC, no methods have been developed that use microbial species or genes as markers for the purposes of screening or risk stratification [38]. In this case-control study, we have examined the association between the presence in stool of a subset of known pro-inflammatory and/or genotoxic bacterial genes and colorectal neoplasia, in samples from two geographically and ethnically distinct populations. This study, even with the limited number of stool samples, revealed a gut bacterial toxin gene profile that was different for each of the two populations (US and PR). Our analyses also revealed that individuals who are positive for multiple bacterial toxin genes have higher odds of developing colorectal neoplasia. Due to the small sample size, most of our observations are not statistically significant, but they reveal a trend that underscores the possibility of incorporating bacterial biomarkers into CRC screening protocols or as tools for risk stratification [38].

In this work, rather than report the detection and prevalence of pro-carcinogenic bacterial species, such as *F. nucleatum* and *B. fragilis*, we screened stool samples for the presence of six specific gut bacterial genes that encode toxins that have been previously shown to promote DNA damage and inflammation [26,28,29,30,32]. Two distinct bacterial toxin gene signatures were observed in stool samples from individuals living in the mainland US and PR. The sample size in these two cohorts (*n* = 30 in US and *n* = 33 in PR) is too small to draw any conclusions regarding how the geographical differences between these groups may influence the gut microbiome. However, dietary patterns are known to shape and influence the gut microbiota, their metabolism, and functional characteristics [39,40,41]. Studies have shown that the dietary patterns of Puerto Rican adults differ from those in the general US population [42]. In addition, host genetics have been reported to modulate the gut microbial composition [43,44,45] and Puerto Rican Hispanics have been shown to have a unique genomic composition [46], which could possibly contribute to the differences observed in their stool bacterial toxin gene profiles compared to the profiles from individuals from the mainland US. Nonetheless, additional studies with a larger number of samples are needed in order to examine the factors that contribute to the observed differences in the gut microbiota in these two populations.

In the samples from the mainland USA, the gene *AMmurB* was found to be associated with CRC. Although not encoding a bacterial toxin itself, this gene is a marker for the presence of *Akkermansia muciniphila*, a mucolytic bacterium whose link to CRC had been previously established [33,47]. It has been thought that the presence of this bacterium in CRC is due to the increased production of the glycoprotein mucin in diseased tissue [33]. This is not surprising since colorectal adenomas have been reported to contain increased levels of certain mucins [48]. Although not reaching statistical significance, the presence of *pks island* was associated with higher odds (OR = 2.7) of having adenomas or CRC. The *pks island* encodes a number of enzymes for the production of a genotoxic natural product colibactin, whose chemical structure and mechanism of action have not been elucidated, but its presence had been previously shown to correlate with CRC and tumor formation [8]. We also report the increased presence of *tcpC* and *usp* in CRC samples (OR > 2.7). The possible role of TcpC in cancer could be related with its known activity as an antagonist of Toll-like receptor 4, an activity that promotes aberrant tissue inflammation [29]. Finally the presence of *usp* in the GI tract had been documented, but its main previously known activity was the promotion of cell cycle arrest and DNA damage in the urinary tract [32]. Although there are a number of plausible mechanisms by which these bacterial genes could favor cancer promotion, we believe that the true clinical significance of these preliminary associations will emerge from validation studies with larger cohorts that more fully reflect the geographical and racial diversity of the US.

In the Puerto Rico samples, stronger associations were observed between the presence of the bacterial toxin genes in our panel and increased odds of having adenomas or CRC. As in the US samples, marginally significant associations were detected between the presence of *pks*, *tcpC*, or *usp*, and colorectal neoplasia. Having 2 or more of any bacterial toxin gene in our panel in stool showed a strong significant association with higher odds of having adenomas. The detection of such a strong significant association within this small sample group warrants further investigation in larger number of individuals and supports that this panel may have potential as a CRC risk stratification tool.

Taken together, our results provide a glimpse into the possible feasibility and clinical utility of a CRC risk stratification and/or screening strategies based on the detection of specific bacterial toxin genes in stool. Although our results need to be further validated in a larger number of patient samples and with more diverse patient populations, it is clear that distinct bacterial toxin gene signatures can be detected and quantified, without having to isolate bacterial clones or to reconstruct species composition. Further research is warranted to evaluate the clinical utility of this PCR-based method in CRC risk stratification and/or screening, and to further explore the mechanisms by which these genes could act as functional effectors in colorectal carcinogenesis.

## Figures and Tables

**Table 1:** A list of six bacterial genes in this study and their known pathogenic mechanism.

Gene Name	Pathogenic Mechanism
*pks island (pks)*	Encodes colibactin, a genotoxin that induces double-strand DNA breaks and genome instability [27,28]
*TIR domain-containing protein (tcpC)*	Toxin modulates host immune response [29]
*gelatinase-E (gelE)*	Pro-inflammatory toxin [30]
*cytotoxic necrotizing factor (CNF)*	Cyclomodulin that promotes proliferation [31]
*uropathogenic specific protein (USP)*	Genotoxin that induces DNA damage [32]
*UDP-N-acetylenolpyruvylglucosamine reductase (murB)*	Nonpathogenic; surrogate marker for *Akkermansia muciniphila*, a mucolytic bacterium associated with CRC [33]

**Table 2:** Gut bacterial toxin gene primer sequences, annealing temperatures, and expected amplicon size for end-point PCR analyses.

Bacterial Gene	Primer Sequence	Annealing Temp (°C)	Size (bp)	Reference
*pks island*	F: GTTTTGCTCGCCAGATAGTCATTC	63	733	Ref. [26]
	R: CAGTTCGGGTATGTGTGGAAGG			
*tcpC*	F: TCGGCGATAGCTTAAGGAGA	56	216	Ref. [26]
	R: CCGCCAAATAATGGCTGTAT			
*gelE*	F: TATGACAATGCTTTTTGGGAT	49	213	Ref. [26]
	R: AGATGCACCCGAAATAATATA			
*cnf-1*	F: AGCGTGCAATCTATCCGTATTT	56	173	Ref. [26]
	R: TGGAATTTCCCCAGTATAGGTG			
*usp*	F: GGTGTTCATACGGGTGAAGG	63	618	This study
	R: CTCAGGGACATAGGGGGAAT			
*AMmurB*	F: GAAATCCGCAGCCATACAAG	57.3	135	This study
	R: CTCCAGAAGACGCTCCATTT			

**Table 3:** Gut bacterial toxin gene primer sequences, annealing temperatures, and expected amplicon sizes for quantitative real-time PCR analyses.

Bacterial Gene	Primer Sequence	Annealing Temp (°C)	Size (bp)	Reference
*pks island*	F: TCGATATAGTCACGCCACCA	63	137	This study
	R: GTCAAGCGAGCATACGAACA			
*tcpC*	F: AGATGGGAGTGGAAGGAGGT	61	144	This study
	R: TGCTTGTAATTTTGCCCAGTC			
*gelE*	F: GGTACAGGCATCTTTGTTGGA	61	131	This study
	R: GCCTCAGAAATTGCCTCCTT			
*cnf-1*	F: AGCGTGCAATCTATCCGTATTT	56	173	Ref. [26]
	R: TGGAATTTCCCCAGTATAGGTG			
*usp*	F: GGTGTTCATACGGGTGAAGG	63	618	This study
	R: CTCAGGGACATAGGGGGAAT			
*AMmurB*	F: GAAATCCGCAGCCATACAAG	57.3	135	This study
	R: CTCCAGAAGACGCTCCATTT			

**Table 4:** Odds ratio (OR) estimation for the association between the presence of gut bacterial toxin genes in stool samples from individuals in the U.S. and colorectal neoplasia (*n* = 30).

	Model 1				Model 2	
Bacterial Gene	Adenoma		CRC		Neoplasia	
	OR (% CI)	*p*-Value	OR (% CI)	*p*-Value	OR (% CI)	*p*-Value
*pks*	2.7 (0.4–29.1)	0.17	2.7 (0.4–19.7)	0.34	3.3 (0.6–19.4)	0.19
*tcpC*	0.5 (0.03–5.9)	0.53	2.7 (0.4–19.7)	0.33	1.3 (0.21–8.5)	0.76
*cnf*	0.5 (0.03–5.9)	0.53	1 (0.1–8.9)	0.99	0.7 (0.1–5.1)	0.73
*usp*	1.6 (0.2–9.9)	0.64	3.5 (0.5–22.3)	0.18	2.3 (0.5–11.7)	0.30
*gelE*	1 (0.2–6.0)	0.99	0.64 (0.1–4.1)	0.64	0.8 (0.2–3.9)	0.79
AMmurB *	9.8 (0.4–219.2)	0.15	14.5 (0.7–316.7)	0.09	11.7 (0.6–228.4)	0.11
≥2 genes	3.2 (0.4–28.8)	0.29	3.7 (0.5–28.5)	0.19	4.4 (0.6–32.5)	0.15

Not having the gene was used as reference. Neoplasia includes adenomas and CRC cases. ORs were estimated through multinomial logistic regression models (Model 1) or logistic regression models (Model 2) where controls were the reference group. * Added 0.05 to each field.

**Table 5:** Odds ratio (OR) estimation for the association between the presence of gut bacterial toxin genes in stool samples from individuals from Puerto Rico and colorectal neoplasia (*n* = 33).

	Model 1				Model 2	
Bacterial Gene	Adenoma		CRC		Neoplasia	
	OR (% CI)	*p*-Value	OR (% CI)	*p*-Value	OR (% CI)	*p*-Value
Pks *	1.6 (0.02–87.8)	0.82	10.0 (0.5–215.8)	0.82	5.4 (0.3–113.7)	0.28
tcpC *	10.4 (0.4–249.0)	0.15	3.5 (0.1–95.1)	0.46	5.4 (0.3–113.7)	0.28
cnf *	0.3 (0.01–6.4)	0.41	1.7 (0.3–10.6)	0.57	1.0 (0.1–13.4)	0.99
*usp*	3.3 (0.4–26.4)	0.26	1.1 (0.1–9.3)	0.93	1.8 (0.2–22.3)	0.84
*gelE*	4.0 (0.3–53.5)	0.30	8.6 (0.8–89.0)	0.07	6.2 (0.6–315.0)	0.16
*AMmurB*	5.5 (0.7–42.6)	0.10	2.8 (0.4–18.9)	0.30	3.5 (0.5–41.3)	0.26
≥2 genes	24.0 (1.1–518.6)	0.04	10.0 (0.9–117.0)	0.07	11.3 (1.0–637.1)	0.05

Not having the gene was used as reference. Neoplasia includes adenomas and CRC cases. ORs were estimated through multinomial logistic regression models (Model 1) or logistic regression models (Model 2) where controls were the reference group. * Added 0.05 to each field.
